# A flexible framework for sparse simultaneous component based data integration

**DOI:** 10.1186/1471-2105-12-448

**Published:** 2011-11-15

**Authors:** Katrijn Van Deun, Tom F Wilderjans, Robert A van den Berg, Anestis Antoniadis, Iven Van Mechelen

**Affiliations:** 1 Center for Computational Systems Biology SymBioSys, Katholieke Universiteit Leuven, 3000 Leuven, Belgium; 2 Department of Psychology, Katholieke Universiteit Leuven, 3000 Leuven, Belgium; 3 GlaxoSmithKline Biologicals, 1330 Rixensart, Belgium; 4 Laboratoire Jean Kuntzmann, Université Joseph Fourier, 38041 Grenoble, France

## Abstract

**1 Background:**

High throughput data are complex and methods that reveal structure underlying the data are most useful. Principal component analysis, frequently implemented as a singular value decomposition, is a popular technique in this respect. Nowadays often the challenge is to reveal structure in several sources of information (e.g., transcriptomics, proteomics) that are available for the same biological entities under study. Simultaneous component methods are most promising in this respect. However, the interpretation of the principal and simultaneous components is often daunting because contributions of each of the biomolecules (transcripts, proteins) have to be taken into account.

**2 Results:**

We propose a sparse simultaneous component method that makes many of the parameters redundant by shrinking them to zero. It includes principal component analysis, sparse principal component analysis, and ordinary simultaneous component analysis as special cases. Several penalties can be tuned that account in different ways for the block structure present in the integrated data. This yields known sparse approaches as the lasso, the ridge penalty, the elastic net, the group lasso, sparse group lasso, and elitist lasso. In addition, the algorithmic results can be easily transposed to the context of regression. Metabolomics data obtained with two measurement platforms for the same set of *Escherichia coli *samples are used to illustrate the proposed methodology and the properties of different penalties with respect to sparseness across and within data blocks.

**3 Conclusion:**

Sparse simultaneous component analysis is a useful method for data integration: First, simultaneous analyses of multiple blocks offer advantages over sequential and separate analyses and second, interpretation of the results is highly facilitated by their sparseness. The approach offered is flexible and allows to take the block structure in different ways into account. As such, structures can be found that are exclusively tied to one data platform (group lasso approach) as well as structures that involve all data platforms (Elitist lasso approach).

**4 Availability:**

The additional file contains a MATLAB implementation of the sparse simultaneous component method.

## Background

The integrated analysis of multiple data sets obtained for the same biological entities under study, has become one of the major challenges for data analysis in bioinformatics and computational biology. Two main causes for this trend are the availability of complementary measurement platforms and the systemic approach to biology; in both cases, multiple data sets are obtained on the same set of samples (e.g., culture samples, tissues). First, examples where several measurement platforms are included are the study of the metabolome composition of *Escherichia coli *(*E. coli*) using several analytical chemical methods to screen for metabolites [1] and the combination of cDNA and Affymetrix chips applied to sixty cancer cell lines [2]. In both examples, there is overlap in the metabolites or genes screened but also complementarity. Second, the modern systemic approach to biology leads to a probing of the biological system on different levels in the cellular organization, such as for example the transcript, protein, and metabolite level [3]. These approaches lead to situations where several data blocks are obtained that are coupled in the sense that they were obtained for the same set of samples. A key issue in integrative data analysis is to analyze such data simultaneously instead of separately or sequentially as this yields an aggregated view. In this respect, simultaneous component methods, that are an extension of principal component analysis (PCA) to the case of multiple coupled data blocks, were proposed and successfully used [4-7].

However, a drawback of component based methods like PCA is their lack of sparseness: Processes underlying the data are revealed by a weighted combination of all variables (these are the genes, transcripts, proteins, metabolites in the aforementioned examples). From an interpretational point of view, this is not very attractive and it also does not reflect that biological processes are expected to be governed by a limited number of genes [8]. The problem holds even more for simultaneous component methods as these involve multiple large sets of variables. To deal with this issue, sparse approaches have been proposed mainly within the context of regression analysis (e.g., [9,10]) but also for principal component analysis [8,11-14]: These select a limited number of variables by shrinking many of the weights to zero which is accomplished by proper penalization of these (regression) weights. A favorable characteristic of such penalty based methods is that the selection is built-in (in contrast to, for example, first filtering and then doing the regression/PCA). Here, we extent sparse PCA to sparse simultaneous component methods, accounting for the fact that the data are structured in several data blocks holding both shared and complementary information. The estimation procedure used is efficient and the associated MATLAB code can be found in the additional file.

First, we present the sparse simultaneous component model, starting from ordinary principal component analysis and sparse PCA. A generic modeling framework is introduced that incorporates several types of penalties. Then we present some results for metabolomics data obtained with two measurement platforms for the same set of *E. coli *samples and we validate the method by means of simulated data.

## Results

### Algorithm

#### Notation

We will make use of the following formal notation: matrices are denoted by bold uppercases, vectors by bold lower case, the transpose by the superscript ^*T*^, and the cardinality by the capital of the letter used to run the index (e.g., this paper deals with *K *data matrices **X**_*k *_with *k *running from 1 to *K*), see [15].

Throughout the paper, we suppose that all variables are mean-centered and scaled to norm one.

#### Model

Simultaneous component analysis is an extension of **principal component analysis (PCA) **to the case of multiple coupled data matrices. Consider the PCA of a single data block **X**_*k *_containing the scores of *I*_*k *_objects (e.g., batches, arrays) on *J*_*k *_variables (e.g., metabolites, genes). In a first model formulation [16] based on component scores, PCA decomposes the data as follows,

(1) $$X_{k}=T_{k}P_{k}^{T}+E_{k}$$

with **T**_*k *_the component scores of the *I*_*k *_objects on the *R *components, **P**_*k *_(of size *J*_*k *_× *R*) the loadings, and **E**_*k *_(of size *I*_*k *_× *J*_*k*_) the matrix of residuals. To identify the model, usually the constraints are imposed that the axes have a principal axes orientation and that the component scores are orthogonal: $T_{k}^{T}T_{k}=I$. Another formulation of the PCA model is based on component weights as follows [17],

(2) $$X_{k}=X_{k}W_{k}P_{k}^{T}+E_{k}$$

with **W**_*k *_(of size *J*_*k *_× *R*) the component weights. Note that we can write **T**_*k *_= **X**_*k*_**W**_*k *_resulting in the equivalence of models (1) and (2). However, usually (2) is constrained to have orthogonal weights: $W_{k}^{T}W_{k}=\text{I}$. Note that under a least squares approach to PCA, **P**_*k *_= **W**_*k *_and thus also $P_{k}^{T}P_{k}=I$. The principal components are interpreted by considering the contribution of the variables to the components. For the score-based model (1) this is based on the fact that the loadings are equal to the correlation of the variables with the components (we suppose the variables to be mean-centered and scaled to norm one). Let **x**_*jk *_be the *j*th variable in data block *k *and **t**_*rk *_the *r*th component for block *k*, then

(3) $$r\left(x_{jk,}t_{rk}\right)=p_{jrk},$$

with *r*(.,.) used as a notation for correlation and *p*_*jrk *_the loading of the *j*th variable on the *r*th component of block *k*. In the weight-based model (2), interpretation of the components is based on the weights as these express each component as a weighted linear combination of the variables,

(4) $$t_{rk}=X_{k}w_{rk}.$$

For both model formulations this implies that for each component a total of *J*_*k *_correlations or weights have to be taken into account in the interpretation. Especially in the case of omics data, that usually consist of thousands of variables, there is a need for methods that facilitate interpretation. To that end, [14] proposed **a sparse PCA method **for the weight based model (2), that shrinks a (large) number of component weights to zero. Their method is based on a least-squares approach to PCA model (2) in which the objective function is augmented with an *l*_1 _penalty (also named lasso) and an $l_{2}^{2}$ (ridge) penalty: Minimize with respect to **W**_*k *_and **P**_*k*_

(5) $$\begin{array}{c} L\left(W_{k},P_{k}\right)=\\ \;\;{∥X_{k}-X_{k}W_{k}P_{k}^{T}∥}^{2}+\lambda_{L}{∥W_{k}∥}_{1}+\lambda_{R}{∥W_{k}∥}_{2}^{2}, \end{array}$$

such that $P_{k}^{T}P_{k}=\text{I}$ and with λ_*L *_≥ 0 and λ_*R *_≥ 0 tuning parameters for the lasso and ridge penalties respectively, ${∥W_{k}∥}_{1}=\sum_{j_{k},r}\left|w_{j_{k}r}\right|$ and ${∥W_{k}∥}^{2}=\sum_{j_{k,}r}w_{j_{k}r}^{2}$. The *lasso*, tuned by the parameter λ_*L*_, has the property to simultaneously shrink coefficients and select variables, keeping only those variables with the highest coefficients. The higher λ_*L*_, the stronger the shrinkage and selection. Note that the selection is done in an unstructured way meaning that correlations between variables are not taken into account. The *ridge *penalty, tuned by λ_*R*_, only shrinks the coefficients and does not perform variable selection (none of the coefficients becomes zero). It is often introduced when it is of interest to group correlated variables [10] or in case of ill-conditioned optimization problems (see [18]) to solve the non-uniqueness of the parameter estimates. A particular case is regression analysis with more variables than objects, *J*_*k *_>*I*_*k*_, which yields an under determined estimation problem. In the context of PCA, this is of relevance for model (5) because the estimation of the component weights boils down to a regression analysis. Adding the ridge penalty with λ_*R *_> 0 solves the non-uniqueness; in addition, with the appropriate normalization, the ridge ensures that the solution of (5) yields the principal components in case λ_*L *_= 0 (see [14]).

The **simultaneous component decomposition **of *K *coupled data blocks **X**_*k *_having a common set of samples (so *I*_1 _= ... = *I*_*k *_= *I*) is given by imposing the constraint that all **T**_*k *_are equal. Applied to the score based model this gives:

(6) $$X_{k}={TP}_{k}^{T}+E_{k},$$

for all *k *and under the constraints of a principal axes orientation and orthogonality of the component scores: **T**^*T*^**T **= **I**. Applying the idea of a common matrix of component scores to the weight based model as used by [14], can be realized as follows,

(7) $$\begin{array}{c} \left[X_{1}\ldots X_{K}\right]=\\ \;\;\;\left[X_{1}\ldots X_{K}\right]{\left[W_{1}^{T}\ldots W_{K}^{T}\right]}^{T}\left[P_{1}^{T}\ldots P_{K}^{T}\right]\\ \;\;\;+\left[E_{1}\ldots E_{K}\right] \end{array}$$

(8) $$=T\left[P_{1}^{T}\ldots P_{K}^{T}\right]+\left[E_{1}\ldots E_{K}\right],$$

under the constraint of a principal axes orientation and orthogonal loadings: $\left[P_{1}^{T}\ldots P_{K}^{T}\right]{\left[P_{1}^{T}\ldots P_{K}^{T}\right]}^{T}=I$. Simultaneous component model (7) shows that the common component scores **T **lie in the space spanned by all variables, this is from all data blocks. For ease of notation, we will use the shorthand notation **X**_*c *_= [**X**_1 _... **X**_*K*_] (of size *I *× Σ_*k *_*J*_*k*_) and $P_{c}={\left[P_{1}^{T}\ldots P_{K}^{T}\right]}^{T}$ and $W_{c}={\left[W_{1}^{T}\ldots W_{K}^{T}\right]}^{T}$ (both of size Σ_*k *_*J*_*k *_× *R*). Note that several simultaneous component models were proposed in the literature: [6] gives an overview that emphasizes the different ways of weighting the data blocks in connection to different principles to realize a fair integration of the data.

The problem that a lot of variables have to be taken into account when interpreting the components is exacerbated in the case of simultaneous component analysis as this involves several blocks of variables. To solve for this problem, we propose to go for a **sparse simultaneous component method **by penalizing either the loadings (in the context of the score based model) or the component weights (in the context of the weights based model) within a least-squares approach. One possibility, in line with sparse PCA, is to use the lasso penalty if necessary in conjunction with a ridge penalty (when grouping of correlated variables is of interest or when Σ_*k *_*J*_*k *_>*I*). However, other types of penalties can be used that, when selecting variables, explicitly take into account that variables belong to (pre-defined) groups/blocks by selecting variables within blocks only, between blocks only (by setting all weights/loadings of an entire block to zero, i.e. dropping an entire group of variables at once), or both within and between blocks. A penalty that introduces selection only within each group is *Elitist lasso *(mixed *l*_1,2 _norm), defined for the *r*th component as

(9) $$\lambda_{E}\underset{k}{\sum }{∥{\text{w}}_{rk}∥}_{1,2}=\lambda_{E}{\underset{k}{\sum }\left(\underset{jk}{\sum }\left|w_{j_{k}rk}\right|\right)}^{2}.$$

Elitist lasso was introduced by [19] in the context of regression analysis. The behavior of this penalty can be understood by observing that it behaves as the lasso within blocks and as the ridge between blocks, resulting in shrinkage and a selection of the variables with the highest coefficients within each block (lasso) and a shrinkage but with no selection between blocks (ridge).

To select entire (pre-defined) groups of variables, the *group lasso *[20] was introduced. It uses the Euclidean norm (also known as a mixed *l*_2,1 _norm; see [19]) of the group coefficients as a penalty,

(10) $$\lambda_{G}\underset{k}{\sum }\sqrt{J_{k}}{∥{\text{w}}_{rk}∥}_{2}=\lambda_{G}\underset{k}{\sum }\sqrt{J_{k}\underset{jk}{\sum }\left(w_{jkrk}^{2}\right).}$$

This penalty behaves as the lasso at the block level and as the ridge within blocks: within blocks shrinkage and grouping of correlated variables occurs however with no selection (behavior of the ridge penalty); between blocks selection of those blocks with the highest sum of squared coefficients occurs while other blocks are dropped (behavior of the lasso). The group lasso applied to groups consisting of one variable only is the same as the lasso. (Note that taking the square root of a squared value is the same as taking the absolute value.) To obtain also sparsity within the groups that are not dropped by the group lasso, [21] proposed the *sparse group lasso *that blends the lasso with the group lasso and implies shrinkage and selection both within and between groups. The behavior of each of the four penalties and associated norms is summarized in Table 1.

**Table 1:** Sparse approaches

Norm	Properties	Sparse approach				
		Lasso	Elastic net	Group lasso	Sparse group lasso	Elitist lasso
*l*_1_	selection and shrinkage at the level of the concatenated data	YES	YES	NO	YES	NO
$l_{2}^{2}$	shrinkage, groups correlated variables	NO	YES	NO	NO	NO
*l*_2,1_	selection and shrinkage of entire blocks	NO	NO	YES	YES	NO
*L*_1,2_	selection and shrinkage within each block	NO	NO	NO	NO	YES

Different norms used in the context of sparse approaches, their properties, and specific sparse approaches based on particular combinations of the penalties. A 'YES' indicates that the norm is active in the approach.

We propose the following **generic functions that combine all penalties**: First, for the approach based on sparse component weights,

(11) $$\begin{array}{c} L\left(W_{k},P_{k}\right)=\\ \;\;\underset{k}{\sum }\left({∥X_{k}-X_{k}W_{k}P_{k}^{T}∥}^{2}+\lambda_{L}{∥W_{k}∥}_{1}\right)\\ \;\;+\underset{k}{\sum }\left(\lambda_{R}{∥W_{k}∥}_{2}^{2}+\lambda_{G}\sqrt{J_{k}}{∥W_{k}∥}_{2}\right)\\ \;\;+\underset{k}{\sum }\left(\lambda_{E}{∥W_{k}∥}_{1,2}\right)\\ \;\;={∥X_{c}-X_{c}W_{c}P_{c}^{T}∥}^{2}+\lambda_{L}{∥W_{c}∥}_{1}\\ \;\;+\lambda_{R}{∥W_{c}∥}_{2}^{2}+\underset{k}{\sum }\left(\lambda_{G}\sqrt{J_{k}}{∥W_{k}∥}_{2}\right)\\ \;\;+\underset{k}{\sum }\left(\lambda_{E}{∥W_{k}∥}_{1,2}\right), \end{array}$$

which has to be minimized with respect to **W**_*k *_and **P**_*c *_under the constraint that $P_{c}^{T}P_{c}=I$. Second, for the approach based on sparse component loadings,

(12) $$\begin{array}{c} L\left(T,P_{k}\right)={∥X_{c}-{TP}_{c}^{T}∥}^{2}+\lambda_{L}{∥P_{c}∥}_{1}+\lambda_{R}{∥P_{c}∥}_{2}^{2}\\ \;\;\;\;\;+\underset{k}{\sum }\left(\lambda_{G}\sqrt{J_{k}}{∥P_{k}∥}_{2}+\lambda_{E}{∥P_{k}∥}_{1,2}\right), \end{array}$$

which has to be minimized with respect to **T **and **P**_*k *_under the constraint that **T**^*T*^**T **= **I**. Note that estimation of the loadings is not a regression problem. Therefore, unlike the model based on sparse weights, unique solutions are obtained when *J*_*k *_>*I*. This is the case even when λ_*R *_= 0.

The generic loss functions (11) and (12) allow for a flexible use of all these approaches to sparseness. All combinations of the four penalties are made possible. However, often some prior idea about the structure (selection within blocks, between blocks, both within and between blocks) exists such that it is not necessary to consider all possible combinations. Furthermore, some combinations are not advisable. For example the combination of the group lasso and elitist lasso does not seem useful because the behavior of the one interferes with the behavior of the other. By setting the appropriate tuning parameters in the objective functions to zero, particular known sparse approaches can be obtained. For example, with λ_*G *_= λ_*E *_= 0 the extension of sparse PCA to simultaneous component analysis is obtained and with λ_*R *_= λ_*E *_= 0 a sparse simultaneous component version of the sparse group lasso in linear regression is obtained. With all four tuning parameters set equal to zero, the ordinary simultaneous component analysis model results. *K *= 1 leads to principal component analysis and setting λ_*G *_= λ_*E *_= 0 yields sparse PCA as proposed by [14]. In Table 1 a summary is given of these different existing sparse approaches in terms of which penalties are active.

#### Algorithm

Given fixed values for the different tuning parameters (λ_*l*_, λ_*R*_, λ_*G*_, and λ_*E*_) and a fixed number of components *R*, we make use of an alternating scheme to minimize (11) or (12) with respect to **W**_*c *_(or **T**) and **P**_*c *_: **W**_*c *_(or **T**) and **P**_*c *_are alternatingly updated, conditional on fixed values for the other parameters. For example, focusing on (11):

• Step 1: Initialize **W**_*c*_

• Step 2: Conditional on the current estimate of **W**_*c*_, obtain the optimal least-squares estimate of **P**_*c *_under the orthogonality constraint as follows (see [22]): **P**_*c *_= **UV**^*T *^with **USV**^*T *^the singular value decomposition of $W_{c}^{T}X_{c}^{T}X_{c}$

• Step 3: Check the stop criteria: 1) Is the difference in loss with the previous iteration smaller than 1*e *- 12 or, 2) is a maximum of 5000 iterations reached? If yes, terminate, and else continue.

• Step 4: Conditional on the current estimate of **P**_*c*_, obtain the update of **W**_*c *_using a majorization minimization procedure (see [23-25] for a general introduction); see the Methods Section for a derivation of the estimate. Return to Step 2.

This particular scheme guarantees that the loss is a non-increasing function of the iterations. Due to the convexity (not strict) and the fact that the loss function is bounded from below by zero, the procedure will converge to a fixed point for suitable starting values. The majorization minimization (MM) procedure has a linear rate of convergence; this slow convergence rate may, however, be compensated for by the efficiency of the calculations (see for example [26]). To account for the problem that the fixed point may represent a local minimum instead of the global optimum, a multistart procedure can be used. See the Methods Section for details on the algorithm used to minimize (12). MATLAB code implementing the algorithms can be found in the supplementary material.

### Testing and implementation

In this section we apply the proposed approach both to empirical and simulated data. The application to empirical data (metabolomics) is mainly for illustrative purposes. The simulated data are used to check how the different penalties (and their interactions) behave under various conditions, and to compare the sparse component weights and sparse component loadings modeling approaches.

#### Metabolomics data

As an illustrative case, we use empirical data on the metabolome composition of 28 samples of *E. coli*. The different samples refer to different environmental conditions and different elapsed fermentation times. Mass spectrometry (MS) in combination with on the one hand gas chromatography (GC) and on the other hand liquid chromatography (LC) as a separation method was used, resulting in two coupled data blocks: a GC-MS block with the peak areas of 144 metabolites in the 28 conditions and a LC-MS block with the peak areas of 44 metabolites in these same conditions. Simultaneous component analysis was previously successfully applied describing the data well by five components (see [5,6]). However, a better understanding of the processes underlying the data may be obtained by a sparse simultaneous component analysis (SCA) approach as this characterizes the components by a few instead of all metabolites and thus facilitates interpretation.

Our proposed method allows to model the data in several ways, depending on the one hand on the choice of penalizing either the weights or the loadings and on the other hand on the particular values of the different tuning parameters. Therefore, we will analyze the data under different options, namely either under model (11) or under model (12) and, for both models, with several combinations of values for the different tuning parameters. Here we explain how we chose a suitable range of values for the tuning parameters using the notation for the model with penalized weights. The different values of λ_*L*_, λ_*G*_, λ_*E*_, and λ_*R *_were chosen in a way that reflects the balance between lack-of-fit and strength of the penalty by setting them as a fraction of ||**X**_*c*_||^2 ^(maximal lack-of-fit) and |**W**_*c*_|_*p*,*q *_with **W**_*c *_obtained from the ordinary SCA solution (maximal value of the penalty). Let λ_*p*,*q *_denote the tuning parameter of the penalty corresponding to the (mixed) *l*_*p*,*q *_norm, then this yields λ_*p*,*q *_= *f*||**X**_*c*_||^2^/|**W**_*c*_|_*p*,*q *_with *f *taking values 0,10^-4^,10^-3^,10^-2^,10^-1^,0.2, 0.5, and 1. We only consider those combinations of non-zero values for the tuning parameters that were considered in the regression literature, namely the lasso, elastic net, group lasso, sparse group lasso, and elitist lasso (see Table 1). Note that the case with all tuning parameters equal to zero corresponds to regular simultaneous component analysis.

First we discuss the results for the approach based on penalized weights, then the approach based on penalized loadings, followed by a brief comparison of the two approaches. We end the empirical application section with a discussion on the choice and interpretation of a particular sparse simultaneous component analysis.

#### Penalized weights

**Figure 1:**
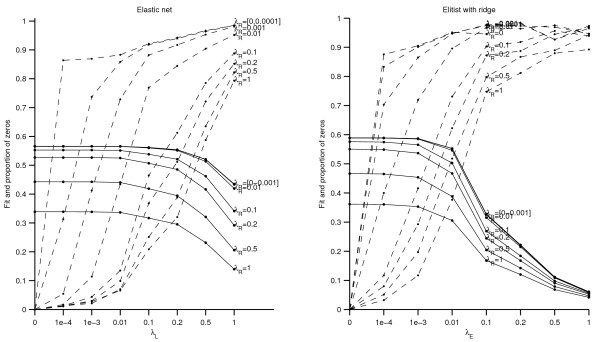
**Adding the ridge panel to the (Elitist) lasso**. Left panel: Elastic net; Right panel: Elitist lasso with ridge penalty. Fit (full lines) and proportion of zeros (dashed lines) in function of the lasso tuning parameter (left panel) and in function of the Elitist lasso tuning parameter (right panel). The different lines refer to different values of the ridge parameter.

**Figure 2:**
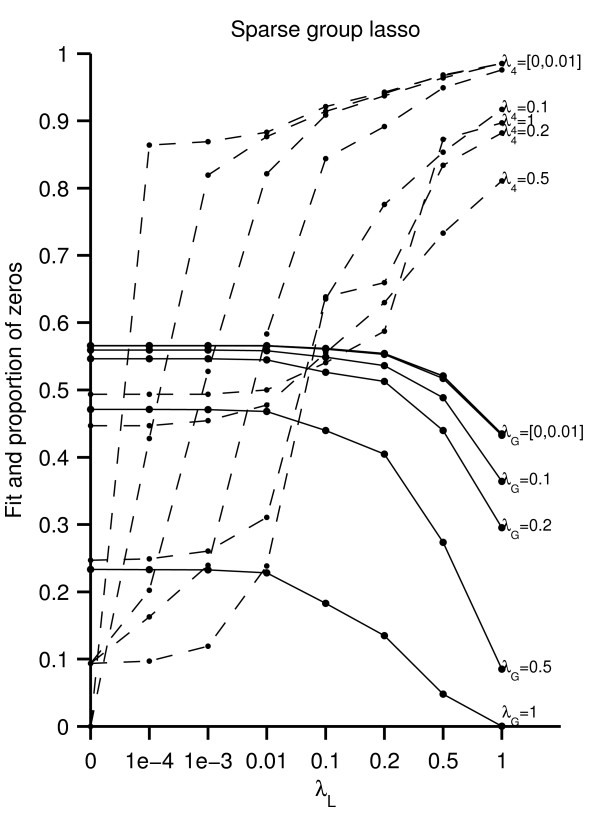
**Sparse group lasso**. Fit (full lines) and proportion of zeros (dashed lines) for the sparse group lasso. The different lines refer to different values of the group lasso penalty.

Table 2 summarizes the results for the approach with a penalty on the component weights and with only one of the tuning parameters different from zero (the ridge penalty on its own is not considered as it does not induce sparsity). Five components are assumed (*R *= 5). For the three resulting types of sparse simultaneous component analyses we report on the one hand the fit of the model to the data and on the other hand the percentage of component weights that are zero. The fit is defined as $1-{∥X_{c}-X_{c}W_{c}P_{c}^{T}∥}^{2}∕{∥X_{c}∥}^{2}$. As could be expected, it holds that increasing the tuning parameter results in a decrease of fit and an increase of the proportion of zero component weights. Comparing the lasso and Elitist lasso, we see that the lasso has a better fit for a similar proportion of zeros which may be attributed to the fact the lasso is less constrained because it does not have to reflect the block structure in the variable selection. Both for the lasso and Elitist lasso the proportion of zeros is very high, even for small values of the tuning parameter. This could be expected as the number of variables is larger than the number of samples and warrants the inclusion of a ridge penalty (see also [14] for the case of the lasso), else non-unique solutions are obtained. Also, at most *I *non-zero weights will be obtained for each component and this may be too sparse, e.g. in the case of micro-array gene expression data obtained for a limited number of (tissue) samples. Such solutions with only *I *non-zero values fit as well as the regular simultaneous component model. To understand this, consider a model with one component: **t **= **Xw **which represents an underdetermined system in case I < Σ_*k *_*J*_*k *_that can be solved exactly by taking only *I *non-zero weights. The group lasso operates at the level of the block and therefore does not show this effect. The effect of adding a ridge penalty to the lasso and elitist lasso is visualized in Figure 1: The higher the value of the parameter that tunes the ridge penalty, the lower the fit and the lower the proportion of zeros. In Figure 2 the results for the sparse group lasso (i.e., combination of lasso and ridge penalty) are summarized. The lines express the fit and the proportion of zero weights in function of the lasso tuning parameter with different lines referring to different values of the group lasso. As illustrated by the figure, there is a qualitative interaction between the two types of penalties in the sense that lower values of the lasso parameter have a strong effect on the number of zeros when the group lasso parameter takes lower values while, conversely, higher values of the lasso parameter have a strong effect when the group lasso parameter takes higher values: As the group lasso shrinks the component weights, the penalty for the lasso becomes lower and hence low values of the lasso tuning parameter are ineffective. Addition of a ridge penalty to the lasso and group lasso parameters may be considered when grouping is important (e.g., as is usual with gene expression data to find modules of co-expressed genes; [27]).

**Table 2:** Summary results for the different simultaneous component analyses with sparse weights

	Lasso		GroupLasso		ElitistLasso	
*f*	Fit	% zeros	Fit	% zeros	Fit	% zeros
0	0.57	0	0.57	0	0.57	0
0.0001	0.57	86	0.57	0	0.57	88
0.001	0.57	87	0.57	9	0.56	92
0.01	0.57	88	0.57	9	0.52	96
0.1	0.56	92	0.56	9	0.26	99
0.2	0.55	94	0.55	25	0.16	97
0.5	0.52	97	0.47	45	0.08	98
1	0.43	99	0.23	50	0.04	99

Different panels correspond to different approaches: The lasso in the left panel, the group lasso in the middle panel, and Elitist lasso in the right panel. Within each panel, both the fit of the model to the data and the percentage of zero weights are reported. The different rows correspond to different values of the tuning parameter.

#### Penalized loadings

A summary of the results obtained for the approach with sparse loadings is given in Table 3. The general result that increasing the tuning parameters yields a decrease in fit and an increase in sparsity also holds here. A comparison between Tables 2 and 3 shows that for an equal proportion of zeros, the fit of models with sparse loadings is (much) lower. This can be understood from the fact that the loadings contribute more directly to the reconstruction of the data than the component weights (compare equations (1) and (2)): for example, in a model with one component, a zero loading results in a zero vector for the reconstructed variable. This also explains why different from the approach based on penalizing the weights with an *L*_1 _penalty, the number of non-zeros is not bounded by *I*. Table 3 also shows that to obtain zero loadings with the group lasso, high values of the tuning parameter are needed.

**Table 3:** Summary results for the different simultaneous component analyses with sparse loadings

	Lasso		GroupLasso		ElitistLasso	
*f*	Fit	% zeros	Fit	% zeros	Fit	% zeros
0	0.57	0	0.57	0	0.57	0
0.0001	0.57	0	0.57	0	0.57	0
0.001	0.57	0	0.57	0	0.57	4
0.01	0.57	0	0.57	0	0.54	19
0.1	0.57	7	0.57	0	0.36	37
0.2	0.56	10	0.56	0	0.28	41
0.5	0.53	20	0.54	0	0.17	47
1	0.46	28	0.46	0	0.10	47

Different panels correspond to different approaches: The lasso in the first panel, the group lasso in the second panel, and Elitist lasso in the last panel. Within each panel, both the fit of the model to the data and the number of zero loadings are reported. The different rows correspond to different values of the tuning parameter.

#### Reflections on penalizing the weights versus the loadings

As illustrated, the results obtained under the model with penalized loadings are different from the results obtained under the model with penalized weights. In our view, the most important differences are at the level of data reconstruction and at the level of interpretation. With respect to data reconstruction, the model based on weights yields a better fit while the model with sparse loadings may yield many zero vectors for the reconstructed data. Also, in this respect, the components based on sparse weights have a higher correlation with the components of the ordinary SCA solution than the components resulting from a model with sparse loadings. With respect to interpretation of the underlying components, for the model based on sparse weights this is done in a regression-like way, while for the model based on sparse loadings it is based on considering loadings as correlations of the variables with the component. In ordinary SCA, the loadings are the correlations and in the sparse model we observed a close connection in that zero loadings represent close to zero correlations and higher loadings represent higher correlations. The weights do not have such a relation with the correlation between the variable and the component.

#### Selection and interpretation of the sparse SCA solution

As has been illustrated in the previous Results Section, the data can be analyzed in many ways depending on choices made with respect to the generic model ((11) or (12)) and with respect to the values of the different tuning parameters. Selection of the appropriate model is of key importance and substantive issues may form a good point of departure. First, concerning the choice of the generic model, the model with penalized weights seems more appropriate for the data at hand because all metabolites can be considered to be involved in the biological processes underlying the data. For applications of component models with sparse loadings to microarray gene expression data, see [28] and [13]. Second, to choose appropriate values for the tuning parameters we consider the properties of the associated penalties. Having components for which the interpretation is tied exclusively to one type of analytical platform (corresponding to the block structure) is convenient. Also, because for each platform many metabolites result, sparseness within each platform/block is needed. This means that we are interested in selection both across and within groups. Recently, there has been a growing interest for methods that perform such a selection [29,30], with particular interest for the group lasso that has been extended and applied in several ways [31-33]. Therefore, we will restrict ourselves to a group lasso type of simultaneous component model, however, including a ridge penalty to account for the fact that grouping is useful (because, within each analytical method, the metabolites belong to several classes of strongly related compounds): λ_*L *_> 0, *λ*_*R *_> 0, λ_*G *_> 0, and *λ*_*L *_= 0. Then, we eliminate solutions that 1) yield components with all weights equal to zero, 2) yield components having non-zero weights for both data blocks, and 3) solutions that do not fit well (fit < .40) or that are not sparse (less than 50 percent of zero weights in a block). The remaining solutions are summarized in Table 4 in terms of the fit and the number of zeros per component. The solutions in bold, with high values for the lasso tuning parameter (*f*_*L *_= 0.5 or 1) and low values for both the ridge and group lasso parameters (*f*_*R *_= 0.0001 and *f*_*G *_= 0.1; or *f*_*R *_= 0.001 and *f*_*G *_= 0.001), show the best tradeoff between fit and sparsity. We select these solutions for an interpretation.

**Table 4:** Overview of fit and sparseness for retained sparse group lasso models

*f*_*L*_	*f*_*R*_	*f*_*G*_	Fit	Number of zeros in				
				C1	C2	C3	C4	C5
**0.5**	**0.0001**	**0.01**	**0.52**	**178**	**176**	**176**	**178**	**179**
0.5	0.0001	0.1	0.49	166	156	167	161	159
0.5	0.0001	0.2	0.44	150	173	145	143	169
0.5	0.001	0.1	0.49	158	167	161	166	156
0.5	0.001	0.2	0.44	169	150	146	145	173
0.5	0.01	0.1	0.48	154	154	166	165	160
1	0.0001	0.01	0.43	184	181	183	184	182
**1**	**0.001**	**0.001**	**0.43**	**181**	**185**	**185**	**185**	**186**
1	0.001	0.01	0.43	181	182	184	183	180
1	0.01	0.0001	0.42	180	183	180	179	174
1	0.01	0.001	0.42	182	179	174	180	179
1	0.01	0.01	0.41	177	180	173	178	181

Solutions with five components that have non-zero component weights in only one data block, a fit > .40, and more than 50 percent of zero weights in the remaining block. The strength of the different tuning parameters is indicated in the first three columns, the fit is displayed in the fourth column, and the 5 remaining columns show for each component (C1-C5) how many of the 188 metabolites received a zero-weight.

The metabolites with non-zero component weights are displayed for both selected solutions in Table 5; Table 6 contains the component scores corresponding to the weights of the solution with *f*_*L *_= 0.5. Observe that the solution with *f*_*L *_= 1 is a further selection of the metabolites in the solution with *f*_*L *_= 0.5. The first component shows an effect of phenyllactate, 3,5-dihydroxypentanoate, and two aromatic amino acids (phenylalanine and tyrosine), together with two branched-chain amino acids (isoleucine and valine); the corresponding component scores (see C1 in Table 6) show a clear increasing linear effect of fermentation time. The second component is made up by metabolites like fumarate, malate, aspartate and are associated to succinate catabolism (see C2 in Table 6) making biological sense as these metabolites are close to succinate in central metabolism. For C3, we find non-zero weights for a large number of (unidentified) disaccharides and pyruvate and lactate and high scores in the oxygen related conditions. The identification of pyruvate and lactate could be indicative of a changing, i.e. reduced, dissolved oxygen concentration in the course of the fermentation as pyruvate can be converted into lactate during anaerobic growth. The fourth component is made up by nucleotides important for the energy metabolism in a cell (i.e. ADP, GDP, UDP) and is associated to the growth condition with an elevated pH at the early (16hrs) phase. Finally, C5 seems specific for the wild type strain, although the relation to the metabolites guanine and thymine (both nucleobases) and the other metabolites is not very clear.

**Table 5:** Metabolites with non-zero weights in the two selected solutions

	metabolite	*f*_*L *_= 0.5	*f*_*L *_= 1
C1	3,5-dihydroxypentanoate:	0.68	0.88
C1	valine:	0.58	0.20
C1	3-phenyllactate or isomer:	0.55	1.21
C1	isoleucine:	0.48	
C1	tyrosine:	0.41	0.03
C1	phenylalanine:	0.40	
C1	unknown mass 304, 319 and 406:	0.01	
C1	spectrum not complete6:	-0.06	
C1	mixed spectrum3:	-0.43	0.36
C1	keto-gluconate (?):	-0.46	0.25
			
C2	fumarate:	1.40	1.99
C2	malate:	0.96	1.06
C2	aspartate:	0.42	
C2	monomethylphosphate:	0.39	
C2	C18:1 fatty acid3:	0.37	0.19
C2	unknown1:	0.37	
C2	spectrum not complete4:	0.20	
C2	mixed spectrum2:	0.19	
C2	glycerate:	0.14	
C2	unknown20:	0.02	
			
C3	lactate:	1.23	2.18
C3	pyruvate:	0.71	0.39
C3	disaccharide12:	0.49	0.11
C3	3-dehydroquinate:	0.38	
C3	disaccharide8:	0.33	
C3	citrate:	0.29	
C3	disaccharide9:	0.27	
C3	unknown mass 318 and 420:	0.17	
C3	unknown mass 217 and 191:	0.17	
C3	disaccharide13:	0.11	
C3	2-hydroxybutanoate:	0.09	
			
C4	ADP:	1.16	1.01
C4	GDP:	0.96	1.21
C4	UDP-glucose:	0.71	0.14
C4	UTP:	0.34	
C4	unknown27:	0.20	
C4	GMP:	0.20	
C4	FBP:	0.09	
			
C5	spectrum not found7:	1.41	2.04
C5	guanine:	0.73	
C5	orotate:	0.51	0.34
C5	spectrum not complete5:	0.31	
C5	mixed spectrum6:	0.24	
C5	N-acetylaspartate		
C5	+ beta-phenylpyruvate:	0.23	
C5	thymine:	0.12	

Metabolites with non-zero component weighs for each of the five components (C1 to C5). The component weights of two selected models are shown that differ in the degree of sparsity (*f*_*L *_= 0.5 and *f*_*L *_= 1).

**Table 6:** Component scores for the selected solution

Condition	Ferm. time	C1	C2	C3	C4	C5
**Reference**	**16**	-0.42	-0.11	-0.06	-0.30	-0.27
	**24**	-0.26	-0.14	0.00	0.29	-0.09
	**32**	0.30	-0.09	-0.26	0.07	0.05
	**40**	0.40	-0.15	-0.27	-0.24	0.03
	**48**	0.38	-0.06	-0.06	0.34	0.09
**pH **+	**16**	-0.35	-0.13	-0.28	**0.99**	-0.25
	**24**	0.08	-0.22	0.14	-0.35	-0.10
	**40**	0.46	-0.20	-0.35	-0.30	-0.13
	**48**	**0.54**	-0.26	-0.38	-0.10	-0.12
oxygen +	**40**	-0.21	0.05	**0.51**	-0.02	-0.13
oxygen ?	**16**	-0.44	-0.24	0.00	-0.24	-0.21
	**24**	-0.22	-0.03	0.42	0.32	-0.15
	**40**	0.34	0.10	**1.05**	0.24	-0.03
	**64**	0.59	0.05	**0.50**	0.24	-0.08
phosphate +	**16**	**-0.54**	-0.23	-0.08	-0.23	-0.23
	**24**	**-0.53**	-0.26	0.18	-0.27	-0.17
	**40**	-0.09	0.06	**0.59**	0.26	-0.10
	**48**	0.14	-0.01	-0.02	0.13	-0.13
phosphate -	**16**	-0.27	-0.25	-0.03	0.04	-0.14
	**24**	0.26	-0.21	0.19	-0.35	0.01
	**40**	**0.53**	-0.21	-0.33	-0.56	-0.14
succinate	**24**	-0.10	**1.03**	-0.09	-0.13	-0.19
	**40**	0.06	**1.21**	-0.13	-0.05	-0.08
	**48**	0.12	**1.07**	-0.11	-0.02	0.19
Wild type	**16**	-0.42	-0.27	-0.34	-0.20	-0.05
	**24**	-0.23	-0.14	-0.31	0.38	0.44
	**40**	-0.11	-0.17	-0.22	0.22	**0.94**
	**48**	-0.04	-0.19	-0.26	-0.14	**1.06**

Component scores for each of the five components (C1-C5). The samples where obtained in a specific environmental condition (first column) and at a particular fermentation time (second column).

#### Simulated data

To validate the proposed sparse simultaneous component method, we make use of simulated data. The general **setup **is that data are generated under some specific conditions and with known structure; after addition of noise, the performance of the method in terms of recovering the underlying structure is assessed. Here, we are particularly interested in two aspects: A first one is whether the penalties reflect the structure in the selection of the variables (i.e., between data blocks; within data blocks; or both between and within data blocks); a second one is the behavior of the method in function of the model (i.e., sparse weights or sparse loadings). We also manipulated the amount of error in the data (5 and 30 percent) and the degree of sparseness (50 and 90 percent of zero weights/loadings). All factors were fully crossed and for each of the resulting 2 × 3 × 2 × 2 = 24 conditions, 5 data sets were generated, resulting in a total of 120 data sets. To obtain a realistic simulation, we **generated the data **using the metabolomics data described in the previous section. 28 samples were sampled with replacement from the original data; then a singular value decomposition was performed to obtain three components: the three loading and weight vectors were obtained as the three right singular vectors corresponding to the three largest singular values and multiplied by these, the three component score vectors were set equal to the corresponding left singular vectors. Sparseness was imposed by setting either weights or loadings equal to zero as follows: In case of sparseness between blocks, all weights/loadings of the first component that correspond to the first data block (the first 144 weights/loadings) were set equal to zero and for the second and third component the weights/loadings corresponding to the second data block (the last 44 weights/loadings) were set equal to zero; in case of sparseness within blocks, 50 or 90 percent of variable indices were randomly sampled and their corresponding weights/loadings were set equal to zero; in case of sparseness within and between data blocks, the two previous strategies were combined. The resulting component loadings and weights were used to generate the true data part using the model part of expressions (1) and (2) (i.e., without the addition of the residual matrices). Noise was then added to this true part of the data with the noise being generated from a normal distribution with mean zero and variance such that these residual matrices account for 5 or 30 percent of the total variation [34]. Each of the data sets was analyzed under both models (sparse weights or sparse loadings) and with varying values for the tuning parameters (*f *equal to 0, 10^-3^, 0.1, 0.5, and 10). The Elitist lasso penalty was only combined with the ridge penalty because it interferes with the lasso and group lasso (see earlier).

**Figure 3:**
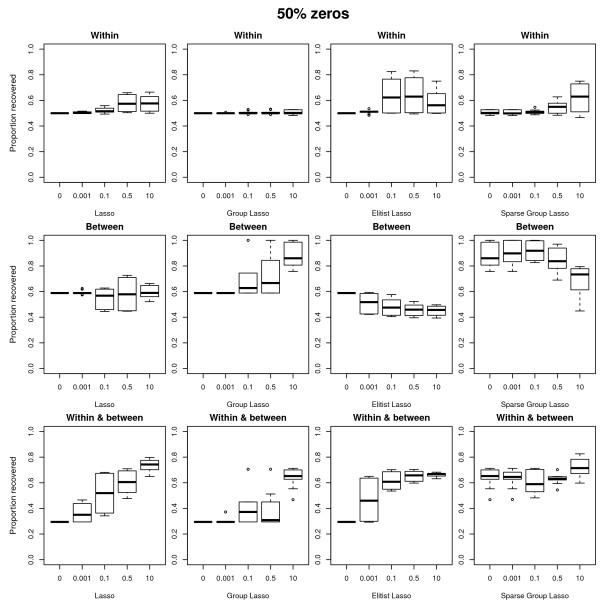
**Boxplots of the proportion of recovered variables: 50 percent of true zeros**. Boxplots of the proportion of variables correctly classified (selected versus dropped) in function of the value of the tuning parameters. Case with 50 percent of the variables dropped. The different panels refer to the different combinations of structure in the variable selection (from top to bottom: within blocks, between blocks, within and between blocks) and of sparseness approach (from left to right: lasso, group lasso, Elitist lasso, and sparse group lasso). The panels referring to the sparse group lasso are with varying values for the lasso tuning parameter and with the group lasso tuning parameter fixed at *f *= 10.

**Figure 4:**
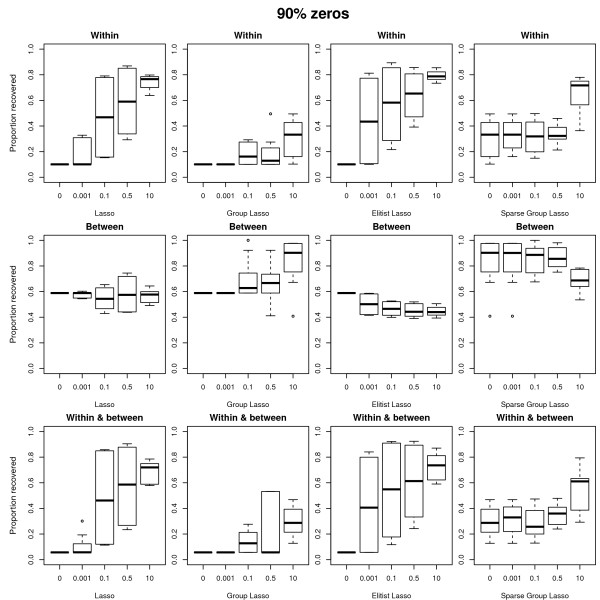
**Boxplots of the proportion of recovered variables: 90 percent of true zeros**. Boxplots of the proportion of variables correctly classified (selected versus dropped) in function of the value of the tuning parameters. Case with 90 percent of the variables dropped. The different panels refer to the different combinations of structure in the variable selection (from top to bottom: within blocks, between blocks, within and between blocks) and of sparseness approach (from left to right: lasso, group lasso, Elitist lasso, and sparse group lasso). The panels referring to the sparse group lasso are with varying values for the lasso tuning parameter and with the group lasso tuning parameter fixed at *f *= 10.

In the **discussion of the results of the simulation study**, we first focus on the conditions where the data are generated and analyzed under the same model (either sparse weights or sparse loadings), the error amounting to 30 percent of the total variation in the data, and the ridge penalty set equal to the smallest non-zero value. Figures 3 and 4 display boxplots of the proportion of variables correctly classified (selected versus dropped) in function of the value of the tuning parameter. Figure 3 refers to the case with 50 percent zero weights/loadings, Figure 4 to the case with 90 percent zero weights/loadings. In each Figure, the different panels refer to the different combinations of structure in the variable selection (from top to bottom: within blocks, between blocks, within and between blocks) and of sparseness approach (from left to right: lasso, group lasso, Elitist lasso, and sparse group lasso). The panels referring to the sparse group lasso are with varying values for the lasso tuning parameter and with the group lasso tuning parameter fixed at *f*_*G *_= 10. In general, the results confirm the expected relation between the structure of the variable selection and the different approaches to sparseness: The best recovery for selection within blocks is by Elitist lasso with a value of 0.5 for the tuning parameter *f*_*E*_, for selection between blocks is by the group lasso with *f*_*G *_= 10, and for selection between and within blocks the sparse group lasso (*f*_*L *_= 0.1 for the lasso). Deviations from the expected behavior occur for the sparse group lasso when selection is both within and between blocks in case of many zeros (see Figure 4): the lasso and Elitist lasso then outperform the group lasso. This can be attributed to the fact that the group lasso is less aggressive than the lasso and Elitist lasso [11]. On the other hand, the lasso and Elitist lasso perform less well when selection is within blocks and the true structure is not so sparse (50 percent of zeros, see the top row of Figure 3) because of their aggressive behavior. Note that a penalty that selects between groups in a more aggressive way was proposed by [11]. The same pattern of results is obtained when the error amounts to 5 percent (though shifted upwards as in these conditions the status of the variables is better recovered) or when the tuning parameter of the ridge penalty takes higher values. In case the ridge equals zero, the box plots show worse results for the lasso and Elitist tuning parameters equal to zero (because there are more variables than objects thus at most 28 non-zero values are obtained for the approach based on sparse weights).

**Figure 5:**
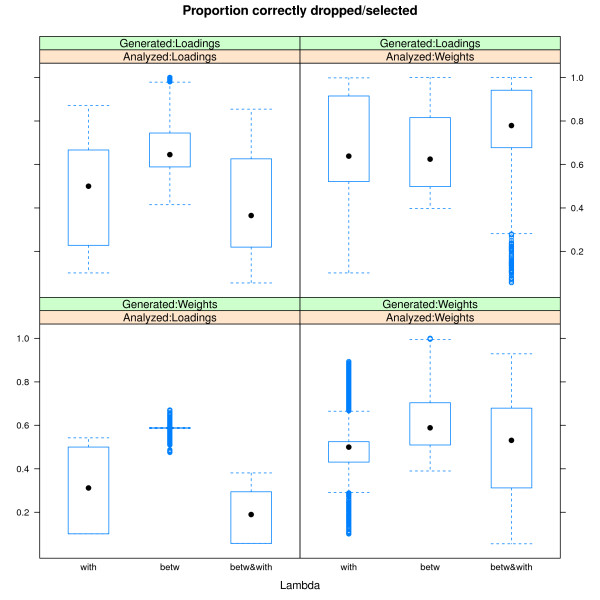
**Comparing sparse weights versus sparse loadings**. Boxplots for the proportion of correctly classified variables. Within panels, the boxplots are displayed in function of the block structure present in the variable selection. The upper panels refer to data generated under a model with sparse loadings, the lower panels to data generated under a model with sparse weights. The panels at the left were obtained when analyzing the data with a model based on sparse weights; the panels at the right with a model based on sparse loadings.

A second point of interest, is the influence of the model used to generate and analyze the data. Figure 5 displays four panels of boxplots for the proportion of correctly classified variables. Within panels, the boxplots are displayed in function of the block structure present in the variable selection. The upper panels refer to data generated under a model with sparse loadings, the lower panels to data generated under a model with sparse weights. The panels at the left were obtained when analyzing the data with a model based on sparse weights and at the right with sparse loadings. In general, analyzing the data with the sparse weights model yields less misclassifications than using the sparse loadings model. However, generating the (underlying) data under a model with sparse weights, in general, results in more misclassifications than generating under a sparse loadings model. These results can be explained by the more direct relation between the loadings and generated or modeled data: Generating the data with sparse loadings imposes a clearer structure than generating them with sparse weights; analyzing/modeling the data with sparse loadings imposes a stronger structure on the modeled data than modeling them with sparse weights. This is because 1) unlike a zero loading, a zero weight for a variable does not necessarily imply a modeled score of zero, because a zero weight for one variable can be compensated by non-zero weights for other variables, and 2) unlike shrinking the weights, shrinking the loadings results more directly in shrunken modeled scores. The latter can be explained by the dependence of the scale of the data, as modeled by PCA model (1), on the scale of the loadings (the model has orthonormal component scores).

## Discussion

We proposed an extension of sparse PCA to the context of several data blocks, relying on a generic modeling framework that allows either for sparse component weights or for sparse component loadings and that incorporates several approaches that were taken to sparsity in the regression literature (including the lasso, elastic net, group lasso, Elitist lasso, and sparse group lasso). A very flexible algorithm was developed that allows to analyze the data under a variety of approaches that take the structure of the data in different ways into account. It also allows for combinations of penalties that were not yet considered in the regression literature.

The flexibility of the approach is important as often a particular kind of structure is needed from data integration methods. The group lasso is a popular tool to find structures that only involve one data block. This is for example relevant in comparative genomics when the focus is on divergence [35] or on tissue-specificity [36]. Elitist lasso, on the other hand, finds sparse structures that involve each of the data blocks. Not only is this of relevance in the aforementioned case of comparative genomics to find conserved processes, but also in a top-down systems biology approach. For example, to integrate microarray gene expression data and interaction data with the aim of finding transcription factors and their target genes [37].

Although the model and algorithm were proposed in the context of simultaneous component analysis, it can be easily translated to the context of principal component analysis and also of regression analysis. In fact the algorithm can be used as it is for PCA and the adaptation to regression analysis is a minor one. In the context of simultaneous component analysis, adaptations of the model (and algorithm) to a context that allows for different values of the tuning parameter for each component and/or each block would be valuable. However, such an extension is not trivial. Moreover, the problem of selecting an optimal model becomes more difficult in that more parameters need to be tuned and this would make the choice of selecting appropriate values for the tuning parameter even more difficult than it already is. A major theoretic challenge for many sparse methods is to find a good method to select the value of the tuning parameters.

## Conclusions

We offered a flexible and sparse framework for data integration based on simultaneous component methods. The method is flexible both with respect to the component model and with respect to the sparse structure imposed: Sparsity can be imposed either on the component weights or on the loadings, and can be imposed either within data blocks, across data blocks, or both within and across data blocks. As such, it allows to find structures exclusively tied to one data platform as well as structures that involve all data platforms. A penalty based approach is used that includes the lasso, the ridge penalty, the group lasso, and Elitist lasso. The method includes principal component analysis, sparse principal component analysis, and ordinary simultaneous component analysis as special cases. Real and simulated data were used to validate the method. We believe the method offers a very flexible and versatile tool for many data integration problems.

## Methods

Here we derive the estimates used in the alternating least squares and iterative majorization algorithm. First, it is shown how the conditional estimates for the objective function relying on sparse component weights can be obtained and then for the objective function relying on sparse loadings.

### Sparse component weights

The generic objective function that we rely on to find a simultaneous component solution with sparse component weights is to minimize

(13) $$\begin{array}{c} L\left(W_{k},P_{k}\right)=\\ \;\;\;\underset{k}{\sum }\left({∥X_{k}-X_{k}W_{k}P_{k}^{T}∥}^{2}+\lambda_{L}{∥W_{k}∥}_{1}+\lambda_{R}{∥W_{k}∥}_{2}^{2}\right)\\ \;\;\;+\underset{k}{\sum }\left(\lambda_{G}\sqrt{J_{k}}{∥W_{k}∥}_{2}+\lambda_{E}{∥W_{k}∥}_{1,2}\right)\\ \;\;\;={∥X_{c}-X_{c}W_{c}P_{c}^{T}∥}^{2}+\lambda_{L}{∥W_{c}∥}_{1}+\lambda_{R}{∥W_{c}∥}_{2}^{2}\\ \;\;\;+\underset{k}{\sum }\left(\lambda_{G}\sqrt{J_{k}}{∥W_{k}∥}_{2}+\lambda_{E}{∥W_{k}∥}_{1,2}\right)\\ \;\;\;=\text{tr}\left[{\left(X_{c}-X_{c}W_{c}P_{C}^{T}\right)}^{T}\left(X_{c}-X_{c}W_{c}P_{c}^{T}\right)\right]\\ \;\;\;+\lambda_{L}{∥W_{c}∥}_{1}+\lambda_{R}{∥W_{c}∥}_{2}^{2}\\ \;\;\;+\underset{k}{\sum }\left(\lambda_{G}\sqrt{J_{k}}{∥W_{k}∥}_{2}+\lambda_{E}{∥W_{k}∥}_{1,2}\right)\\ \;\;\;=\text{tr}\left[X_{c}^{T}X_{c}-2X_{c}^{T}X_{c}W_{c}P_{c}^{T}+P_{c}W_{c}^{T}X_{c}^{T}X_{c}W_{c}P_{c}^{T}\right]\\ \;\;\;+\lambda_{L}{∥W_{c}∥}_{1}+\lambda_{R}{∥W_{c}∥}_{2}^{2}\\ \;\;\;+\underset{k}{\sum }\left(\lambda_{G}\sqrt{J_{k}}{∥W_{k}∥}_{2}+\lambda_{E}{∥W_{k}∥}_{1,2}\right)\\ \;\;\;=\text{tr}X_{c}^{T}X_{c}-2\text{tr}X_{c}^{T}X_{c}W_{c}P_{c}^{T}+\text{tr}P_{c}W_{c}^{T}X_{c}^{T}X_{c}W_{c}P_{c}^{T}\\ \;\;\;+\lambda_{L}{∥W_{c}∥}_{1}+\lambda_{R}{∥W_{c}∥}_{2}^{2}\\ \;\;\;+\underset{k}{\sum }\left(\lambda_{G}\sqrt{J_{k}}{∥W_{k}∥}_{2}+\lambda_{E}{∥W_{k}∥}_{1,2}\right), \end{array}$$

with respect to **W**_*c *_and **P**_*c *_and under the constraint $P_{c}^{T}P_{c}=I$. λ_*L*_, λ_*R*_, λ_*G*_, and λ_*E *_are considered to be known non negative constants. We use an alternating approach in which each set of parameters is updated in turn while keeping the remaining sets fixed on their last update. Let **P**_*c *_be the first set to be updated, conditionally upon fixed values for **W**_*c*_. Rewriting (13) gives

(14) $$\begin{array}{c} L\left(W_{c},P_{c}\right)=k_{1}-2\text{tr}W_{c}^{T}X_{c}^{T}X_{c}P_{c}\\ \;\;\;\;\;+\text{tr}P_{c}^{T}P_{c}W_{c}^{T}X_{c}^{T}X_{c}W_{c} \end{array}$$

with $k_{1}=X_{c}^{T}X_{c}+\lambda_{L}{∥W_{c}∥}_{1}+\lambda_{R}{∥W_{c}∥}_{2}^{2}+\sum_{k}\left(\lambda_{G}\sqrt{J_{k}}{∥W_{k}∥}_{2}+\lambda_{E}{∥W_{k}∥}_{1,2}\right)$ the terms that are constant with respect to **P**_*c*_. Using $P_{c}^{T}P_{c}=I$ yields

(15) $$L\left(W_{c},P_{c}\right)=k_{2}-2\text{tr}W_{c}^{T}X_{c}^{T}X_{c}P_{c}$$

with $k_{2}=k_{1}+\text{tr}W_{c}^{T}X_{c}^{T}X_{c}W_{c}$. The minimization of (15) under the constraint of orthogonal loadings is equivalent to the maximization of $\text{tr}W_{c}^{T}X_{c}^{T}X_{c}P_{c}$ under the same constraint. This is a problem with known closed form solution [22]

(16) $$P_{c}=VU^{T}$$

with **U **and **V **the left and right singular vectors of $W_{c}^{T}X_{c}^{T}X_{c}$.

The minimization of (13) with respect to **W**_*c *_is not a standard problem due to the Lasso, Group Lasso, and Elitist Lasso penalties on **W**_*c*_. We will make use of a numerical procedure, known as Majorization Minimization (MM) or also Iterative Majorization, which has been proven to be a superior algorithmic strategy in regularization problems [25,38]. Briefly stated, MM replaces functions that are complicated to minimize by surrogate functions that are easy to minimize, that lie on/above the original function, and that touch the original function in the so-called supporting point. These properties lead to the sandwich inequality [23].

A useful property of majorizing functions is that a sum of terms can be majorized by majorizing the terms [39]. Therefore, a majorizing function can be obtained for (13) by finding a linear or quadratic majorizing function for each of the penalty terms except the ridge. First we consider the Lasso penalty: $\lambda_{L}{∥W_{c}∥}_{1}=\sum_{hk,r,k}\lambda_{L}\left|w_{j_{k}r_{k}}\right|$. Applying the additivity property again, we need to find a majorizing function for $\left|w_{j_{k}r_{k}}\right|$. Such a function is [40]

(17) $$\left|w_{j_{k}r_{k}}\right|\leq \frac{1}{2}\frac{w_{j_{k}r_{k}}^{2}}{\left|w_{j_{k}r_{k}}^{o}\right|}+\frac{1}{2}\left|w_{j_{k}r_{k}}^{o}\right|,$$

with $w_{j_{k}r_{k}}^{o}$ the current estimate of $w_{j_{k}r_{k}}$ that was obtained in the previous iteration. This yields

(18) $$\begin{array}{ll} \lambda \underset{jk,r,k}{\sum }\left|w_{j_{k}rk}\right| & \leq \lambda \underset{jk,r,k}{\sum }\left(\frac{1}{2}\frac{w_{j_{k}r_{k}}^{2}}{\left|w_{j_{k}rk}^{o}\right|}+\frac{1}{2}\left|w_{j_{k}rk}^{o}\right|\right)\;\\ & =\frac{\lambda }{2}\text{Vec}{\left(W_{c}\right)}^{T}D_{1}\;\text{Vec}\left(W_{c}\right)+k_{3},\;\\ & \; \end{array}$$

with the Vec notation indicating that the matrix is vectorized, with $k_{3}=\sum_{j_{k},r,k}\frac{\lambda }{2}\left|w_{j_{k}rk}^{o}\right|$, and with **D**_1 _a diagonal matrix containing the ${\left|w_{j_{k}rk}^{o}\right|}^{-1}$ on its diagonal. Second, we consider the *k *Group Lasso penalty terms $\lambda_{G}{\left|W_{k}\right|}_{2}=\lambda_{G}{\left(\sum_{j_{k},r}w_{j_{k}r}^{2}\right)}^{1∕2}$. A majorizing function for the root is (see [39])

(19) $$\begin{array}{c} \lambda_{G}\underset{k}{\sum }{\left(\underset{j_{k},r}{\sum }w_{j_{k}r}^{2}\right)}^{1∕2}\leq \\ \;\;\frac{\lambda_{G}}{2}\underset{k}{\sum }{\left({\underset{j_{k},r}{\sum }\left(w_{j_{k}r}^{o}\right)}^{2}\right)}^{1∕2}\\ \;\;+\frac{\lambda_{G}}{2}\underset{k}{\sum }{\left(\underset{j_{k},r}{\sum }{\left(w_{j_{k}r}^{o}\right)}^{2}\right)}^{-1∕2}\left(\underset{j_{k},r}{\sum }w_{j_{k}r}^{2}\right)\\ \;=k_{4}+\frac{\lambda_{G}}{2}\text{Vec}{\left(W_{c}\right)}^{T}D_{2}\text{Vec}\left(W_{c}\right), \end{array}$$

with $k_{4}=\frac{\lambda_{G}}{2}\sum_{k}{\left(\sum_{j_{k},r}{\left(w_{j_{k}r}^{o}\right)}^{2}\right)}^{1∕2}$, and with **D**_2 _a diagonal matrix containing the ${\left(\sum_{j_{k},r}{\left(w_{j_{k}r}^{o}\right)}^{2}\right)}^{-1∕2}$ on its diagonal. Third, we majorize the Elitist Lasso penalty term $\lambda_{E}{\left|W_{k}\right|}_{1,2}=\lambda_{E}{\left(\sum_{j_{k},r}\left|w_{j_{k}r}\right|\right)}^{2}$ with the following quadratic function (see [39]),

(20) $$\begin{array}{c} \lambda_{E}\underset{k}{\sum }{\left(\underset{j_{k},r}{\sum }w_{j_{k}r}^{2}\right)}^{1∕2}\leq \\ \;\;\lambda_{E}\underset{k}{\sum }\left(\left(\underset{j_{k},r}{\sum }\left|w_{j_{k}r}^{o}\right|\right)\underset{j_{k},r}{\sum }\frac{w_{j_{k}r}^{2}}{\left|w_{j_{k}r}^{o}\right|}\right)\\ \;=k_{5}\text{ + }\lambda_{E}\text{Vec}{\left(W_{c}\right)}^{T}D_{3}\text{Vec}\left(W_{c}\right), \end{array}$$

with **D**_3 _a diagonal matrix containing the $\left(\sum_{j_{k,}r}\left|w_{j_{k}r}^{o}\right|\right){\left(\left|w_{j_{k}r}^{o}\right|\right)}^{-1}$ on its diagonal.

Combining (13) with the results (18), (19), and (20), we obtain the following majorizing function for (13):

(21) $$\begin{array}{c} L\left(W_{c},P_{c}\right)=\\ \;\;\;{∥X_{c}-X_{c}W_{c}P_{c}^{T}∥}^{2}+\lambda_{L}{∥W_{c}∥}_{1}+\lambda_{R}{∥W_{c}∥}_{2}^{2}\\ \;\;\;+\underset{k}{\sum }\left(\lambda_{G}{∥W_{k}∥}_{2}+\lambda_{E}{∥W_{k}∥}_{1,2}\right)\\ \;={∥\text{Vec}\left(X_{c}\right)-\text{Vec}\left(X_{c}W_{c}P_{c}^{T}\right)∥}^{2}\\ \;\;\;+\lambda_{L}{∥W_{c}∥}_{1}+\lambda_{R}{∥W_{c}∥}_{2}^{2}\\ \;\;\;+\underset{k}{\sum }\left(\lambda_{G}{∥W_{k}∥}_{2}+\lambda_{E}{∥W_{k}∥}_{1,2}\right)\\ \;\leq {∥\text{Vec}\left(X_{c}\right)-\left(P_{c}⊗X_{c}\right)\text{Vec}\left(W_{c}\right)∥}^{2}\\ \;\;\;+\text{Vec}{\left(W_{c}\right)}^{T}\left(D_{sup}\right)\text{Vec}\left(W_{c}\right)+k\\ \;=Q\left(W_{c},P_{c}\right), \end{array}$$

with $D_{sup}=\frac{\lambda_{L}}{2}D_{1}+\frac{\lambda_{G}}{2}D_{2}+\lambda_{E}D_{3}+\lambda_{R}\text{I},I$, an identity matrix, and *k *= *k*_3 _+ *k*_4 _+ *k*_5_. This function can be minimized with respect to **W**_*c *_by finding the value for which the partial derivative of (21) is zero. The partial derivative equals

(22) $$\begin{array}{c} \frac{\partial Q}{\partial {\text{W}}_{c}}=\\ \;-2{\left(P_{c}⊗X_{c}\right)}^{T}\left[\text{Vec}\left(X_{c}\right)-\left(P_{c}⊗X_{c}\right)\text{Vec}\left(W_{c}\right)\right]\\ \;+2\left(D_{sup}\right)\text{Vec}\left(W_{c}\right), \end{array}$$

and is equal to zero for

(23) $$\begin{array}{c} \text{Vec}\left(W_{c}\right)=\\ \;\;\;{\left[D_{sup}+{\left(P_{c}⊗X_{c}\right)}^{T}\left(P_{c}⊗X_{c}\right)\right]}^{-1}{\left(P_{c}^{T}⊗X_{c}\right)}^{T}\text{Vec}\left(X_{c}\right)\\ \;=\;\;\;{\left[D_{sup}+\text{I}⊗\left(X_{c}^{T}X_{c}\right)\right]}^{-1}\text{Vec}\left(X_{{}_{c}}^{T}X_{c}P_{c}\right), \end{array}$$

where the inverse is taken of a block-diagonal matrix. **W**_*c *_is obtained by rearranging Vec(**W**_*c*_). Note that the second derivative is positive so (23) is a minimum of (21). In this equation, the penalty terms occur as diagonal matrices that are summed together in the matrix **D**_*sup *_and with the variance-covariance matrix of the data; the resulting matrix is inverted and will be dominated by large values on the diagonal (yielding small values after inversion). This shows the behavior of the penalties: increasing the tuning parameters results in such large diagonal values; furthermore, the diagonal matrices themselves are inverse functions of the weights in the previous iteration of the algorithm such that small weights further enhance the shrinkage or selection. Note that the matrix to be inverted in equation (23) is of the form **D **+ **A**^*T*^**A **with **D **a diagonal matrix; then, the following holds [41],

(24) $${\left(D+A^{T}A\right)}^{-1}=D^{-1}-D^{-1}A^{T}{\left(I+XD^{-1}X^{T}\right)}^{-1}AD^{-1}$$

which may be useful when *J*_*k *_>*I*.

### Sparse loadings

The generic objective function that we rely on to find a simultaneous component solution with sparse component weights is to minimize

(25) $$\begin{array}{c} L\left(T,P_{k}\right)=\\ \;\;\;\underset{k}{\sum }\left({∥X_{k}-{TP}_{k}^{T}∥}^{2}+\lambda_{L}{∥P_{k}∥}_{1}+\lambda_{R}{∥P_{k}∥}_{2}^{2}\right)\\ \;\;\;+\underset{k}{\sum }\left(\lambda_{G}\sqrt{J_{k}}{∥P_{k}∥}_{2}+\lambda_{E}{∥P_{k}∥}_{1,2}\right)\\ \;={∥X_{c}-{TP}_{c}^{T}∥}^{2}+\lambda_{L}{∥P_{c}∥}_{1}+\lambda_{R}{∥P_{c}∥}_{2}^{2}\\ \;\;\;+\underset{k}{\sum }\left(\lambda_{G}\sqrt{J_{k}}{∥P_{k}∥}_{2}+\lambda_{E}{∥P_{k}∥}_{1,2}\right)\\ \;=\text{tr}\left[{\left(X_{c}-{TP}_{C}^{T}\right)}^{T}\left(X_{c}-{TP}_{c}^{T}\right)\right]\\ \;\;\;+\lambda_{L}{∥P_{c}∥}_{1}+\lambda_{R}{∥P_{c}∥}_{2}^{2}\\ \;\;\;+\underset{k}{\sum }\left(\lambda_{G}\sqrt{J_{k}}{∥P_{k}∥}_{2}+\lambda_{E}{∥P_{k}∥}_{1,2}\right)\\ \;=\text{tr}\left[X_{c}^{T}X_{c}-2X_{c}^{T}{TP}_{c}^{T}+P_{c}T^{T}{TP}_{c}^{T}\right]\\ \;\;\;+\lambda_{L}{∥P_{c}∥}_{1}+\lambda_{R}{∥P_{c}∥}_{2}^{2}\\ \;\;\;+\underset{k}{\sum }\left(\lambda_{G}\sqrt{J_{k}}{∥P_{k}∥}_{2}+\lambda_{E}{∥P_{k}∥}_{1,2}\right)\\ \;=\text{tr}X_{c}^{T}X_{c}-2\text{tr}X_{c}^{T}{TP}_{c}^{T}+\text{tr}P_{c}{TP}_{c}^{T}\\ \;\;\;+\lambda_{L}{∥P_{c}∥}_{1}+\lambda_{R}{∥P_{c}∥}_{2}^{2}\\ \;\;\;+\underset{k}{\sum }\left(\lambda_{G}\sqrt{J_{k}}{∥P_{k}∥}_{2}+\lambda_{E}{∥P_{k}∥}_{1,2}\right), \end{array}$$

with respect to **T **and **P**_*k *_under the constraint that **T**^*T*^**T **= **I**. λ_*L*_, λ_*R*_, λ_*G*_, and λ_*E *_are considered to be known non negative constants. In case all tuning parameters are equal to zero, a regular simultaneous component analysis results and in that case the algorithm should be based on SVD of the concatenated data. We use an alternating approach in which each set of parameters is updated in turn while keeping the remaining sets fixed on their last update. Let **T **be the first set to be updated, conditionally upon fixed values for **P**_*c*_. Rewriting (25) gives

(26) $$L\left(T,P_{c}\right)=k_{6}-2\text{tr}X_{c}^{T}{TP}_{c}^{T}$$

with $k_{6}=\text{tr}\left[X_{c}^{T}X_{c}+P_{c}P_{c}^{T}\right]+\lambda_{L}{∥P_{c}∥}_{1}+\lambda_{R}{∥P_{c}∥}_{2}^{2}+\sum_{k}\left(\lambda_{G}\sqrt{J_{k}}{∥P_{k}∥}_{2}+\lambda_{E}{∥P_{k}∥}_{1,2}\right)$ the terms that are constant with respect to **T**. Minimizing function (26) is equivalent to maximizing $\text{tr}P_{c}^{T}X_{c}^{T}T$ with known closed form solution [22]

(27) $$T=VU^{T}$$

with **U **and **V **the left and right singular vectors of $P_{c}^{T}X^{T}$.

Combining (25) with the results (18), (19), and (20) adapted to the case of loadings, we obtain the following majorizing function for (25):

(28) $$\begin{array}{c} L\left(T,P_{c}\right)=\\ \;\;\;{∥X_{c}-{TP}_{c}^{T}∥}^{2}+\lambda_{L}{∥P_{c}∥}_{1}\\ \;\;\;+\lambda_{R}{∥P_{c}∥}_{2}^{2}+\underset{k}{\sum }\left(\lambda_{G}{∥P_{k}∥}_{2}+\lambda_{E}{∥P_{k}∥}_{1,2}\right)\\ \;={∥\text{Vec}\left(X_{c}\right)-\text{Vec}\left({TP}_{c}^{T}\right)∥}^{2}+\lambda_{L}{∥P_{c}∥}_{1}\\ \;\;\;+\lambda_{R}{∥P_{c}∥}_{2}^{2}+\underset{k}{\sum }\left(\lambda_{G}{∥P_{k}∥}_{2}+\lambda_{E}{∥P_{k}∥}_{1,2}\right)\\ \;\leq {∥\text{Vec}\left(P_{c}\right)-\left(I⊗T\right)\text{Vec}\left(P_{{}_{c}}^{T}\right)∥}^{2}\\ \;\;\;+\text{Vec}{\left(P_{c}\right)}^{T}\left(D_{sup}\right)\text{Vec}\left(P_{c}\right)+k\\ \;=Q\left(T,P_{c}\right), \end{array}$$

and the first derivative of *Q*(**T**, **P**_*c*_) with respect to **P**_*c *_is equal to zero for

(29) $$\begin{array}{c} \text{Vec}\left(P_{c}^{T}\right)=\\ \;\;\;\;{\left[D_{sup}+{\left(I^{T}⊗T\right)}^{T}\left(I^{T}⊗T_{c}\right)\right]}^{-1}{\left(I^{T}⊗T\right)}^{T}\text{Vec}\left(X_{c}\right)\\ \;\;=\;\;\;{\left[D_{sup}+I\right]}^{-1}\text{Vec}\left(T^{T}X_{c}\right). \end{array}$$

## List of abbreviations

*E. coli: Escherichia coli *GC: Gas Chromatography; LC: Liquid Chromatography; MM: Majorization Minimization; MS: Mass Spectrometry; PCA: Principal Component Analysis; SCA: Simultaneous Component Analysis; SVD: Singular Value Decomposition;

## Authors' contributions

KVD derived and implemented the algorithms, performed the data analysis, and drafted the manuscript. TFW participated in the data analysis, model selection, and simulation study. RvdB carried out the interpretation of the results and helped to draft the manuscript. AA and IVM conceived of the study. All authors read and approved the final manuscript.

## Supplementary Material

Additional file 1 **MATLAB code**. The zip file SparseSCA.zip contains four MATLAB functions and a script (test script.m) to illustrate the use of the main function (sparsesca weights.m). The main functions sparsesca weigths.m and sparsescaloadings.m implement the proposed sparse simultaneous component algorithms. Click here for file
